# Supplementary material for: Global hypo-methylation in a proportion of glioblastoma enriched for an astrocytic signature is associated with increased invasion and altered immune landscape
Source: eLife. 2022 Nov 22;11:e77335. doi: 10.7554/eLife.77335 (PMC9681209; doi:10.7554/eLife.77335)
Supplement: Figure 2—source data 1. [file elife-77335-fig2-data1.zip › Figure_2_source_data_1/Figure_2I_J/homerResults/motif23.similar.html]

motif23

## Information for motif23

A
G
C
T
C
G
A
T
C
G
A
T
A
G
C
T
G
C
A
T
A
G
C
T
C
G
T
A
A
C
T
G
C
G
A
T
C
T
G
A
  
Reverse Opposite:  

G
A
C
T
C
G
T
A
T
G
A
C
A
C
G
T
C
T
G
A
C
G
T
A
C
T
G
A
C
G
T
A
G
C
T
A
C
T
G
A
  

|  |  |
| --- | --- |
| p-value: | 1e-32 |
| log p-value: | -7.596e+01 |
| Information Content per bp: | 1.735 |
| Number of Target Sequences with motif | 40.0 |
| Percentage of Target Sequences with motif | 5.51% |
| Number of Background Sequences with motif | 1.7 |
| Percentage of Background Sequences with motif | 0.62% |
| Average Position of motif in Targets | 88.7 +/- 53.8bp |
| Average Position of motif in Background | 84.3 +/- 30.4bp |
| Strand Bias (log2 ratio + to - strand density) | 0.3 |
| Multiplicity (# of sites on avg that occur together) | 1.05 |
| Motif File: | file (matrix) reverse opposite |

### Similar de novo motifs found

|  |  |  |  |  |  |  |  |
| --- | --- | --- | --- | --- | --- | --- | --- |
| Rank | Match Score | Redundant Motif | P-value | log P-value | % of Targets | % of Background | Motif file |
| 1 | 0.676 | C T A G G T A C G C T A G A T C G C A T C G T A C G A T T A C G C T G A T C G A G T C A C G T A | 1e-23 | -54.900042 | 5.79% | 1.01% | motif file (matrix) |
| 2 | 0.638 | C A G T A T G C G A C T A G C T G A C T G T A C T C A G G C A T | 1e-14 | -34.272636 | 6.89% | 1.79% | motif file (matrix) |
| 3 | 0.735 | C A G T A C G T A G C T A C G T C G A T G A C T G C T A C T A G C G A T C G T A A C G T A C G T A G C T A G T C G T C A | 1e-10 | -23.885435 | 3.58% | 0.75% | motif file (matrix) |
